# Overexpression of PtVDL1 in *Phaeodactylum tricornutum* Increases Fucoxanthin Content under Red Light

**DOI:** 10.4014/jmb.2309.09018

**Published:** 2023-10-20

**Authors:** Seungbeom Seo, Kwang Suk Chang, Min Sun Choi, EonSeon Jin

**Affiliations:** 1 Department of Life Science, Research Institute for Natural Sciences, Hanyang University, Seoul 04763, Republic of Korea; 2 Korea Radio-Isotope Center for Pharmaceuticals, Korea Institute of Radiological and Medical Sciences, Seoul 01812, Republic of Korea

**Keywords:** *Phaeodactylum tricornutum*, PtVDL1, fucoxanthin, overexpression, transformation, red light

## Abstract

*Phaeodactylum tricornutum* is a model diatom with significant biotechnological applications, including enhancing biomass, biofuel, and carotenoid production. Specifically, owing to the capacity of this organism to serve as a valuable source of essential raw materials for pharmaceuticals and nutraceuticals, ongoing research is actively focused on enhancing its productivity. One of the genes involved in various stages of fucoxanthin (Fx) biosynthesis, violaxanthin de-epoxidase like 1 (VDL1), has recently been identified. To validate the intracellular function of this gene and boost Fx production through overexpression, we established and examined three transgenic *P. tricornutum* lines characterized by elevated P. tricortunum VDL1 ( PtVDL1) expression and evaluate their cell growth and Fx productivity. These transgenic lines exhibited substantially increased PtVDL1 mRNA and protein levels compared to the wild type (WT). Notably, the enzyme substrate violaxanthin was entirely depleted and could not be detected in the transformants, whereas it remained at constant levels in the WT. Interestingly, under standard white light conditions, Fx productivity in the transformants remained unchanged; however, but after 48 h of exposure to red light, it increased by up to 15%. These results indicate that PtVDL1-overexpressing *P. tricornutum* has industrial potential, particularly for enhancing Fx production under red light conditions.

## Introduction

Brown unicellular diatoms, belong to the Bacillariophyceae, are mainly found in phytoplankton species, which are microscopic eukaryotes. They play a significant role in carbon fixation through photosynthesis and are responsible for approximately 20% of the primary global production [1]. The annual production of organic carbon by marine diatoms through photosynthesis is comparable to that of all terrestrial rainforests [2]. As a result, microalgae, including marine diatoms, are recognized as resources for mitigating the accumulation of carbon dioxide, which causes global warming [3]. Marine diatoms are vital primary producers in aquatic food chains. In addition to their ecological role, they are useful organisms for industrial applications seeking to exploit their high productivity. The use of biomass from highly productive diatoms could be an attractive option for the production of valuable raw materials such as pharmaceuticals, nutraceuticals, and biofuels. Diatoms are beneficial organisms for investigating their ecological relevance and potential industrial applications [4, 5].

Carotenoids are essential components of photosynthetic organisms, that help cells absorb light efficiently and protect them from photo-oxidative damage [6, 7]. Their antioxidant properties make them increasingly valuable for sustainable commercial use. Fucoxanthin (Fx) is a commercial carotenoid commonly found in marine brown algae and diatoms. Carotenoids are highly valuable and are associated with a variety of health benefits, such as antioxidant, anti-cancer and anti-obesity agent [8, 9]. Although brown seaweed and some microalgae can produce Fx, diatoms typically exhibit higher Fx production than other producers [10]. Diatoms have been reported to have Fx contents ranging 0.57-59.2 mg/gram of dry weight, depending on the algal strain, culture conditions, culture scale, and extraction methods [11]. Numerous studies have explored various culture methods and conditions to enhance Fx content and productivity. Maximizing Fx production requires the optimization of the cell culture environment. Productivity enhancement methods have yielded successful results in determining cell growth stages for harvesting; varying Fx content under different light intensities and quality conditions; varying culture conditions in nutritional modes, such as autotrophy or mixotrophy; and altering the nitrate supply environment [12].

To utilize diatoms for commercial production, it is necessary to increase the Fx content and productivity with additional efforts. *P. tricornutum* is a typical marine diatom that contains a high amount of Fx and is used as a model organism for investing the biosynthetic pathway of Fx and improving production [11, 13]. The entire genome of *P. tricornutum* has been sequenced [14] and can be genetically modified using several molecular tools [15, 16]. Because of these advantages, researchers have been studying *P. tricornutum* to determine the Fx biosynthetic pathway in diatoms and increase its Fx contents and productivity.

**Fig. 1:**
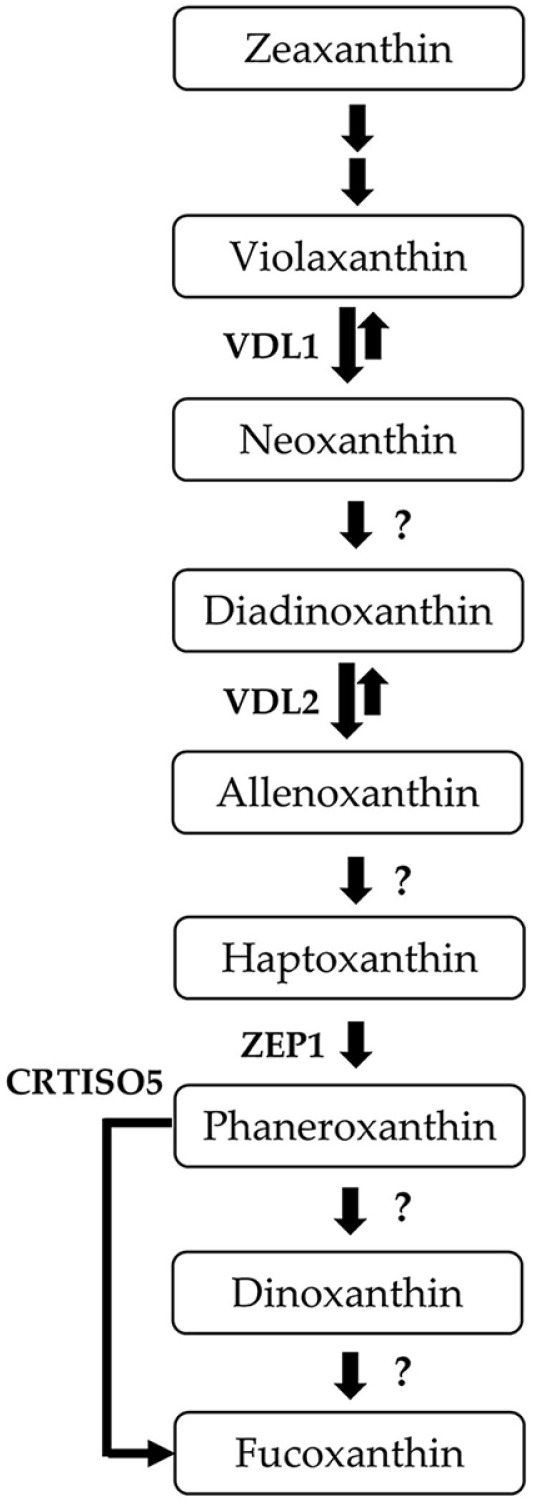
Suggested pathway of fucoxanthin biosynthesis in diatoms, in accordance with recent research [13, 30]. This pathway starts from zeaxanthin. Question marks indicate unknown enzymes. VDL, violaxanthin de-epoxidase-like; ZEP, zeaxanthin epoxidase; CRTISO5, a family of carotenoid isomerases.

The initial pathways of carotenoid biosynthesis in plants and microalgae are well-understood [17]. Commencing from the phytoene precursor C20 geranylgeranyl pyrophosphate, b-carotene is synthesized in a series of steps, followed by zeaxanthin-to-violaxanthin conversion through the violaxanthin cycle. Although recent studies have provided insights into the conversion of violaxanthin to Fx, the complete biosynthetic pathway remains unclear (Fig. 1). It is anticipated that violaxanthin can be converted into neoxanthin or diadinoxanthin, both of which can be further converted into Fx. However, the specific enzymes involved in these conversions have not yet been fully elucidated. Several studies have suggested that the modification of genes thought to be involved in the Fx biosynthetic pathway results in altered Fx production [17].

The violaxanthin cycle is facilitated by zeaxanthin epoxidase (ZEP), a key player in the conversion of zeaxanthin into violaxanthin through the intermediate antheraxanthin. Its antagonistic violaxanthin de-epoxidase (VDE) induces the production of zeaxanthin to dissipate excess chloroplast energy during high light stress, whereas ZEP stimulates violaxanthin production when the light intensity is reduced. Analysis of the genome of the diatom *P. tricornutum* revealed multiple violaxanthin de-epoxidase-like (VDL) genes whose specific functions have not been fully elucidated [13]. However, recent studies have identified that the violaxanthin de-epoxidase-like 1 (VDL1) protein catalyzes the conversion of violaxanthin to neoxanthin, the early product of Fx biosynthesis [18].

In the present study, to enhance Fx production, we overexpressed a gene involved in the biosynthetic pathway of Fx and examined its characteristics. We overexpressed endogenous PtVDL1 in *P. tricornutum* and characterized the transformants. We investigated whether overexpression of PtVDL1 could increase the conversion of violaxanthin to neoxanthin and determined the conditions under which this increase in Fx content could be achieved. Furthermore, we confirmed an increase in Fx content according to changes in culture conditions.

## Materials and Methods

### Culture Conditions

Axenic cultures of *P. tricornutum* Bohlin (CCMP632) were purchased from the National Center for Marine Algae and Microbiota (NCMA) at the Bigelow Laboratory for Ocean Sciences (USA). The cells were cultivated in f/2-Si medium (ASW) supplemented with 40 mM Tris–hydrochloric acid (HCl), pH 7.2, at 20°C under a 12-h light/12-h dark cycle. Culture flasks were agitated at 130 rpm using an orbital shaker. White light (~100 μmol m^−2^ s^−1^) was provided by fluorescent lamps, and red light was obtained from filtered light using red cellophane papers. In addition, 10 mM NaHCO_3_ was added to the culture medium to enhance cell growth.

### Construction of the Transformation Vector for VDL1 Overexpression

To validate the VDL1 sequences from *P. tricornutum* and construct the transformation vector, CDS sequences were amplified from wild-type (WT) cDNA using Pfu DNA polymerase (ELPIS-Biotech, Korea). The PCR product was cloned directly into the T-Blunt vector using the T-Blunt PCR Cloning kit (SolGent, Korea). To detect VDL1 protein expression by western blotting, a c-myc tag was fused to the C-terminus of VDL1. The pPhaT-BP vector, as previously reported [15], containing the Fx chlorophyll a/c–binding protein B (fcpB) promoter from *P. tricornutum*, was used for VDL1 overexpression. The *Shble* gene was excised from the vector and replaced with the N-acetyltransferase (NAT) gene.

### Generation of Transgenic Overexpressing VDL1

Washed M17 tungsten particles (1.1 μm in diameter) were coated with the transformation vector, and 5×10^7^ *P. tricornutum* cells were loaded onto f/2-Si 1.2% agar plates. Particle bombardment was performed using a Biolistic Particle Delivery System PDS-1000/He (Bio-Rad Laboratories, USA) fitted with 1550 psi rupture discs, following the manufacturer's instructions. The gene transfer protocol was adapted from that of Falciatore *et al*.[19]. Bombarded cells were spread onto F/2 (50% SW) agar plates containing 150 μg/ml Nourseothricin, and resistant colonies appeared after 3–4 weeks. Colonies were transferred to fresh agar plates and screened using colony PCR. Cells were suspended in distilled water and used as templates for PCR with rTaq 5×PCR Master Mix (ELPIS-Biotech, Korea).

### Quantitative Reverse Transcription (qRT)-PCR and Western blotting

RNA extraction and quantitative RT-PCR were performed as previously described [15]. Data are presented as the mean of two technical replicates for each independently prepared biological sample (n=3), with standard deviation (SD). PtVDL1 expression levels were normalized to the expression of the gene encoding TATA-box binding protein (TBP) as an internal reference (Table S1).

Protein samples were prepared by harvesting the cells through centrifugation at 2,000 ×*g* for 15 min. Pellets were suspended in extraction buffer (10 mM Tris–HCl, 1 mM EDTA, 0.2% sodium dodecyl sulphate (SDS), and 1× protease inhibitor cocktail; Thermo Scientific, USA). The cells were disrupted by sonication (four times for 30 s each). The total extracted protein was quantified using the Pierce bicinchoninic acid (BCA) protein assay kit (Thermo Scientific) and loaded (20 μg per lane) onto a 10% SDS-polyacrylamide gel electrophoresis (PAGE) gel for separation. Separated proteins were electrotransferred onto a polyvinylidene fluoride (PVDF) membrane for immunoblotting. The primary antibody used was an Myc Tag Polyclonal Antibody (Invitrogen, USA) at a dilution of 1:2500, and the secondary antibody was used at a dilution of 1:20000.

### Southern Blotting

For Southern blot analysis, genomic DNA was prepared from the WT and transformants. Five micrograms (5 μg) of genomic DNA, digested with the restriction enzyme EcoRI, was separated on a 0.8% agarose gel and transferred onto a Hybond-N+ membrane (GE Healthcare, USA). To prepare the probe, the NAT in pPhaT-BP-VDL1 was amplified using PCR using specific primers. Probes were labeled according to the manufacturer’s instructions using Amersham AlkPhos Direct Labeling Reagents (GE Healthcare) and hybridized probes were detected using Amersham CDP-Star Detection Reagents (GE Healthcare).

### Estimation of Cell Growth and Measurement of Biomass

The batches were inoculated at a concentration of 10^6^ cells/ml using freshly cultivated cells. Cell growth was quantified by counting cells using a Neubauer cell-counting chamber. Cultures were harvested employing 1.2-μm Isopore membrane filters (RTTP; Merck Millipore, IRL). Ten milliliters of each culture were subjected to filtration. Subsequently, the filters were air-dried within a 65°C chamber for 24 h and then weighed. The weights of the dried cells were then determined. To accurately determine the Fx content of *P. tricornutum*, two parameters were estimated from the same culture volume: cell-based Fx content and dry cell weight-based Fx content.

### Pigment Analysis Using High-Performance Liquid Chromatography (HPLC) and LC-QTOF/MS

Pigments were extracted from the harvested cells by mixing them with 100% methanol and vigorously vortexing for 5 min. The mixture was then centrifuged at 4°C for 20 min, and the resulting supernatant was filtered through a 0.2 μm nylon filter. HPLC analysis was conducted using a Shimadzu Prominence HPLC model LC-20AD equipped with Waters Spherisorb 5.0 μm ODS1 4.6 × 250 mm cartridge column. The filtered extract was subjected to HPLC analysis, according to established protocols [20].

Additionally, pigment analysis using LC-QTOF/MS was conducted on an Agilent 1290 series binary pump system coupled with an AB Sciex TripleTOF 5600 plus mass spectrometer equipped with an electrospray ionization source. Chromatographic separation occurred on a Poroshell 120 EC-C18 column (2.7 μm, 3.0 × 100 mm; Agilent Technologies) with the column oven temperature maintained at 30°C. The mobile phases for HPLC consisted of 0.1% formic acid in distilled water (solvent A) and 0.1% formic acid in acetonitrile (solvent B). The elution program was as follows: an initial gradient of 70–90% solvent B over 0–5 min, maintained for 1.5 min, followed by a 4.4-min re-equilibration to the initial conditions over 0.1 min, all at a flow rate of 0.4 ml/min. Mass detection was performed in positive ion mode with the following parameters: curtain gas at 25 psi, dry temperature at 500°C, ion spray voltage at 4500 V, and helium as the collision gas.

## Results

### Construction of Transformants Overexpressing PtVDL1

To interpret the putative role of PtVDL1 in the Fx biosynthesis-related genes in *P. tricornutum*, we assessed the gene encoding VDL1 (EEC48043) based on genomic sequencing and a previous study [18]. We cloned the open reading frame of PtVDL1 from *P. tricornutum* CCMP632, and the candidate gene of PtVDL1 showed similarity of 99% (450/453 amino acids) to that of PHATRDRAFT_46155 (GenBank XP_002180635) protein in *P. tricornutum* CCAP 1055/1 (Fig. S1).

**Fig. 2:**
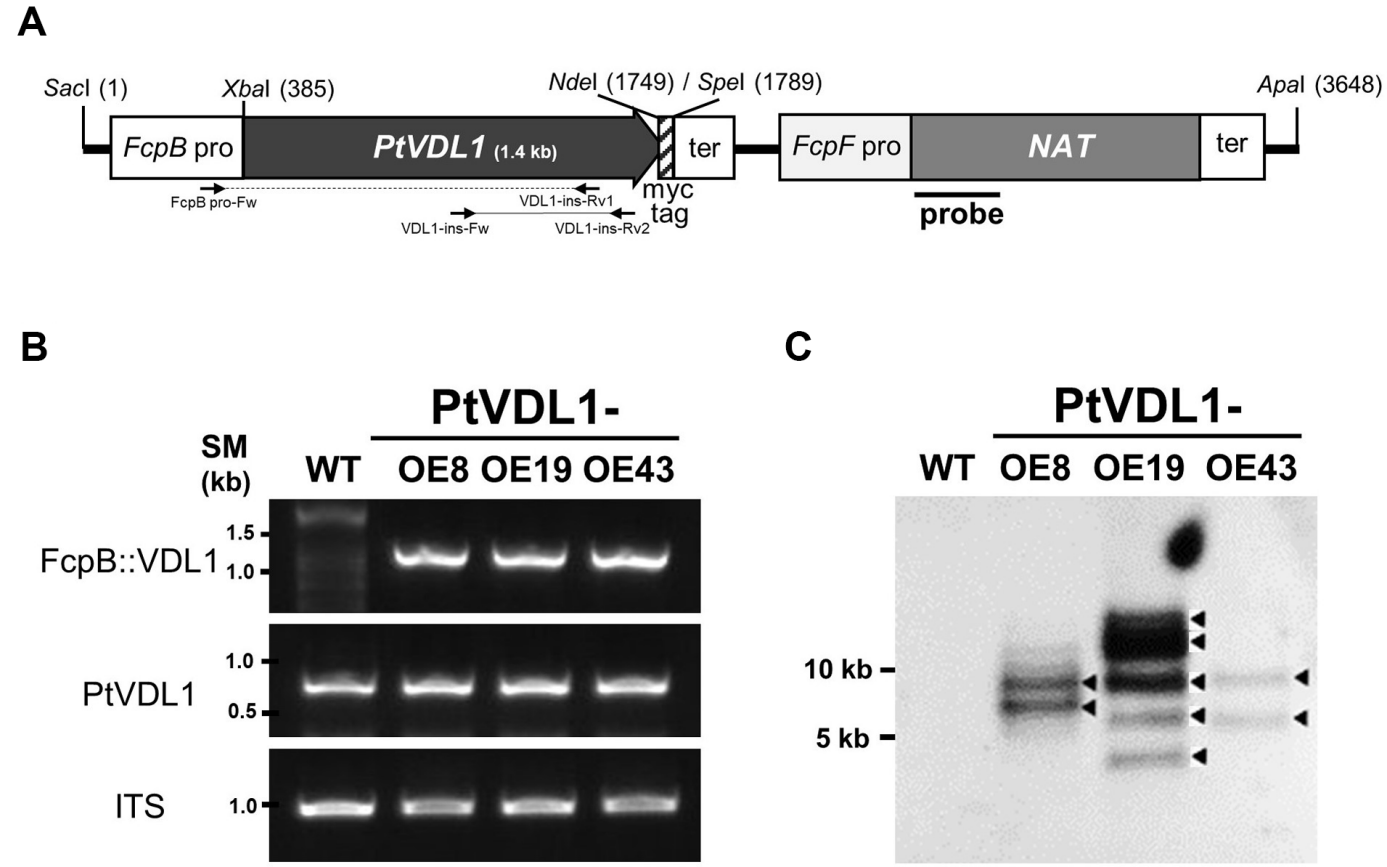
Generation of transgenic overexpressing PtVDL1 and genomic analysis. (**A**) The schematic map of PtVDL1 overexpression cassette and NAT expression cassette in transformation vector. (**B**) Genomic PCR of the wild-type (WT) and PtVDL1-overexpressing strains (PtVDL1-OE8, -OE9, -OE43); Upper: PCR for FcpB::VDL1 with primers of FcpB pro-Fw and VDL1-ins-Rv (underlined in Fig. 2A); Middle: PCR for VDL1 with primers of VDL1-iFw and VDL1-iRv (underlined in Fig. 2A); Bottom: PCR for internal transcribed spacer (ITS) (1.1 kb); SM, DNA size marker; (**C**) Southern blot analysis of WT and PtVDL1-overexpressing strains. Genomic DNA (5 μg) was digested with EcoRI. The partial DNA fragment of NAT was used as a probe (S1).

The zeocin-selectable marker downstream of the Fx chlorophyll a/c binding protein F (fcpF) promoter was replaced with the nourseothricin NAT gene in the previously constructed vectors to modify the pPhat-BP vector [15]. Additionally, we constructed a PTVDL1 overexpression vector by fusing the fcpB promoter upstream of PtVDL1 and placing the fcpA terminator downstream. The myc tag was designed to be expressed in a fused form at the C-terminal end of PtVDL1 (Fig. 2A). The transformation vector constructed to overexpress PtVDL1 was transformed into *P. tricornutum*. Transgenic cells were selected on selective medium containing nourseothricin sulfate. PCR analysis of the selected colonies was performed using primers flanking the fcpB promoter and PtVDL1 gene-specific primers to distinguish between endogenous and exogenous PtVDL1 genes and to verify the integration of the exogenous fcpB::PtVDL1-myc tag cassette. Among the putative transformants grown on the screening medium, we collected 48 colonies and confirmed insertion of the exogenous PtVDL1 gene. 1.2 kb PCR products obtained using genomic DNA confirmed the successful integration of the exogenous PtVDL1 gene into 21 colonies. The PCR analysis results are presented for three representative transformants in Fig. 2B. However, a foreign gene was not detected in the WT strain. Furthermore, the presence of endogenous PtVDL1 and internal transcribed spacer (ITS) genes was confirmed in both WT and transgenic lines (Fig. 2B). To determine the number of copies of the exogenous PtVDL1 gene present in the putative transformants, Southern blot analysis was performed using the NAT gene fragment as a probe (underlined in Fig. 2A). In the WT, no band was detected; however, in each of the three transformants, the following was observed: at least two bands in transformants PtVDL1-OE8 and 43, and at least five bands in OE19 (Fig. 2C). These results confirm that at least two exogenous PtPEPC1 genes were stably integrated into the genome of the three selected transformants.

### Overexpressing PtVDL1 in the Transgenic Lines

**Fig. 3:**
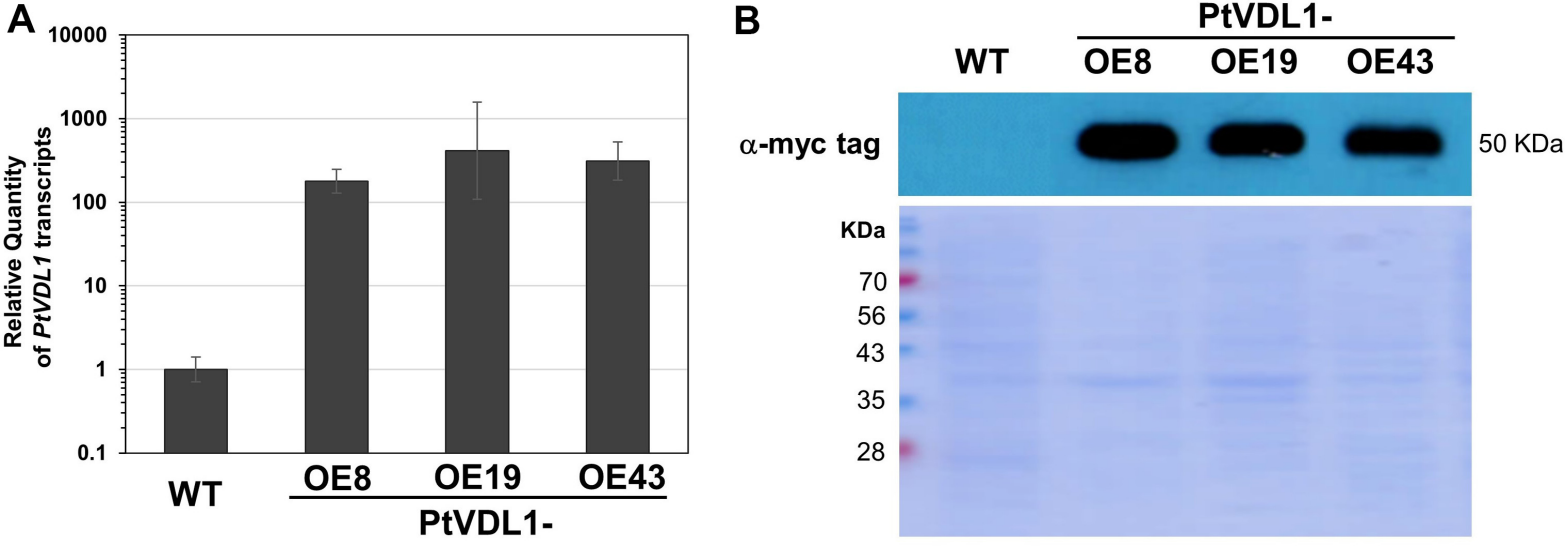
Transcriptional and translational analysis of PtVDL1 overexpression in *P. tricornutum*. (**A**) Relative mRNA quantity of PtVDL1 in the wild-type (WT) and transgenic strains (PtVDL1-OE8, -OE9, -OE43). Error bars represent the mean value from three independent experiments. Statistically significant differences were determined by Student’s *t*-test (***p<0.001, **p<0.01, *p<0.05). (**B**) Immunoblotting with anti-myc tag for analysis of overexpression of PtVDL1 (fused with myc-tag epitope) in transgenic strains. SDS-PAGE gel image is shown as a loading control.

The relative expression levels of PtVDL1 transcript were quantified using quantitative RT-PCR in the WT and three transgenic cell lines during the late exponential phase after 5 d of subculture. PtVDL1 exhibited significantly high quantitative expression, with the relative abundance of the transcript being approximately 180-, 420-, and 310-fold greater in PtVDL1-OE8, OE19, and OE43 transgenic cells, respectively, than in the WT PtVDL1 transcript (Fig. 3A). These results demonstrate that the levels of PtVDL1 transcript were significantly higher in vivo in the three transgenic lines than in the WT.

To determine whether the increase in PtVDL1 transcript levels resulted in an increase in PtVDL1 protein levels, we evaluated the expression of a fusion protein of exogenous PtVDL1 and the myc tag by immunoblot analysis using an anti-myc antibody. Upon analysis, the antibody identified a band of approximately 50 kDa that corresponded to the estimated molecular weight of the fused PtVDL1 and c-myc tag. This band was not visible in WT *P. tricornutum* but was extremely intense in all three transformants. A Coomassie-stained gel containing 20 mg of total protein per lane is also shown below as an immunoblotting result (Fig. 3B). This confirmed that the myc-fused form of PtVDL1 was consistently expressed at high levels in PtVDL1-OE lines. This confirmed the uniform amount of protein and high levels of expression of the myc-fused form of the VDL1 protein in the PtVDL1-OE lines. This demonstrates that PtVDL1 was transcriptionally and translationally upregulated in all three transformants.

### Changes in the Content of Violaxanthin in the PtVDL1 OE Lines

**Fig. 4:**
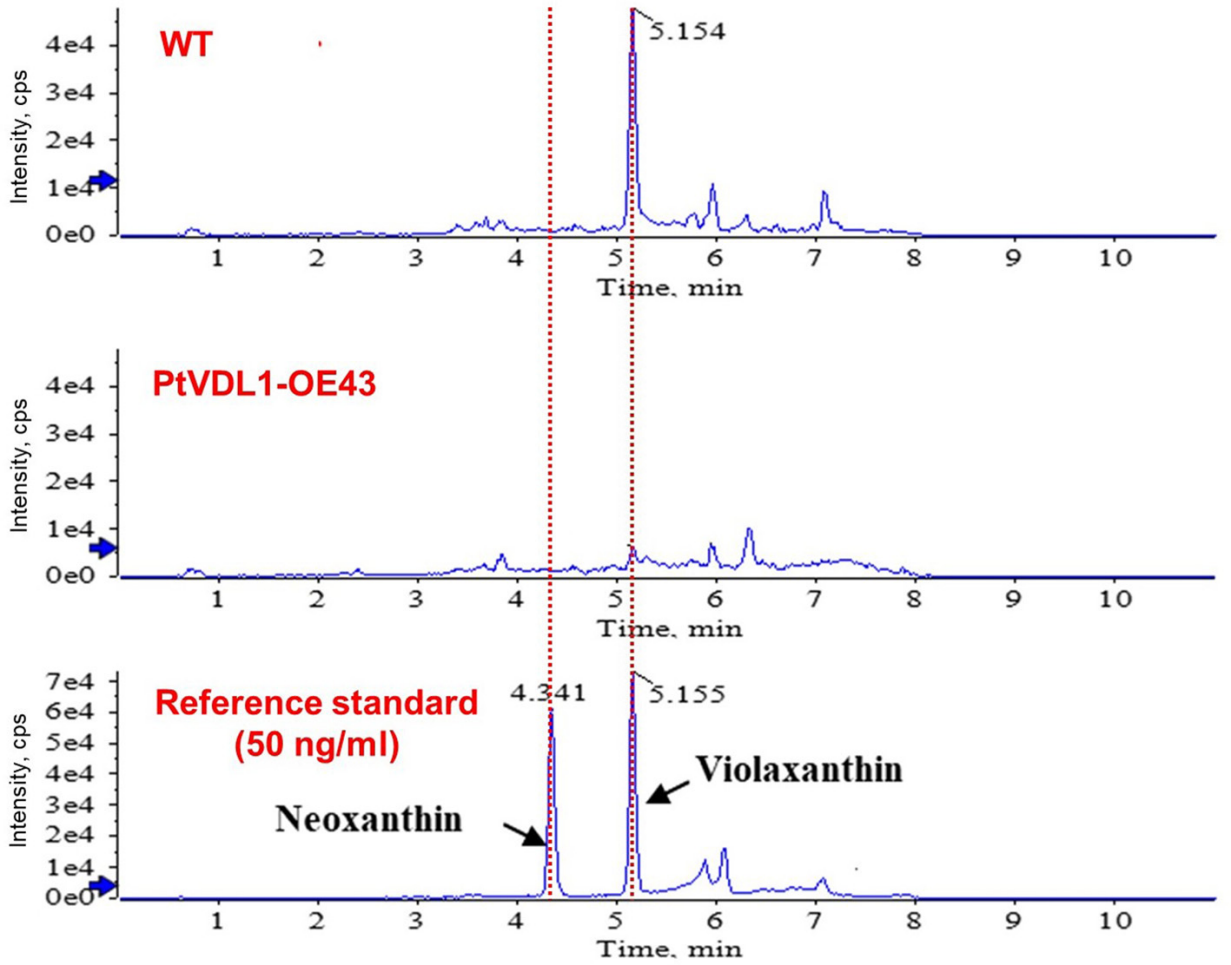
Analysis of neoxanthin and violaxanthin contents in WT and overexpression lines of PtVDL1. Neoxanthin and violaxanthin contents in WT and PtVDL1-OE43 are analyzed using LC-QTOF/MS. Reference standards represent the retention time of each pigment. Neoxanthin = 4.34, Violaxanthin = 5.16.

The functional characterization of PtVDL1 from *P. tricornutum* was confirmed through transient transformation of tobacco leaves with this gene, which exhibited a decrease in violaxanthin content with the accumulation of neoxanthin [18]. Therefore, we aimed to confirm this functional characterization by overexpressing PtVDL1. HPLC analysis using LC-QTOF/MS was performed to determine the changes in the endogenous content of neoxanthin and violaxanthin in *P. tricornutum*. First, reference standard samples of neoxanthin and violaxanthin (50 ng/ml) were used to determine retention times of 4.34 and 5.16 min, respectively (Fig. 4).

To ascertain whether overexpression of the VDL1 protein caused changes in the levels of violaxanthin and neoxanthin in carotenoid biosynthesis, we evaluated the levels of these two carotenoids in the control and overexpression transformants. Violaxanthin, a substrate of VDL1, was also detected in the WT. However, neoxanthin, a product of this enzymatic reaction, was not detected. In contrast, violaxanthin, the major natural substrate of VDL1, was not present in the overexpressing lines with increased PtVDL1 protein expression (Table 1, Fig. 4). It is speculated that the neoxanthin produced is used as an intermediate in Fx biosynthesis and is continuously utilized in the production of the final Fx; therefore, it is not detected in vivo. These results indicate that the overexpression of PtVDL1 in *P. tricornutum* may promote the biosynthesis of Fx by enhancing the consumption of violaxanthin.

### Effect of Overexpressed PtVDL1 on Cell Growth and Fx Production

**Fig. 5:**
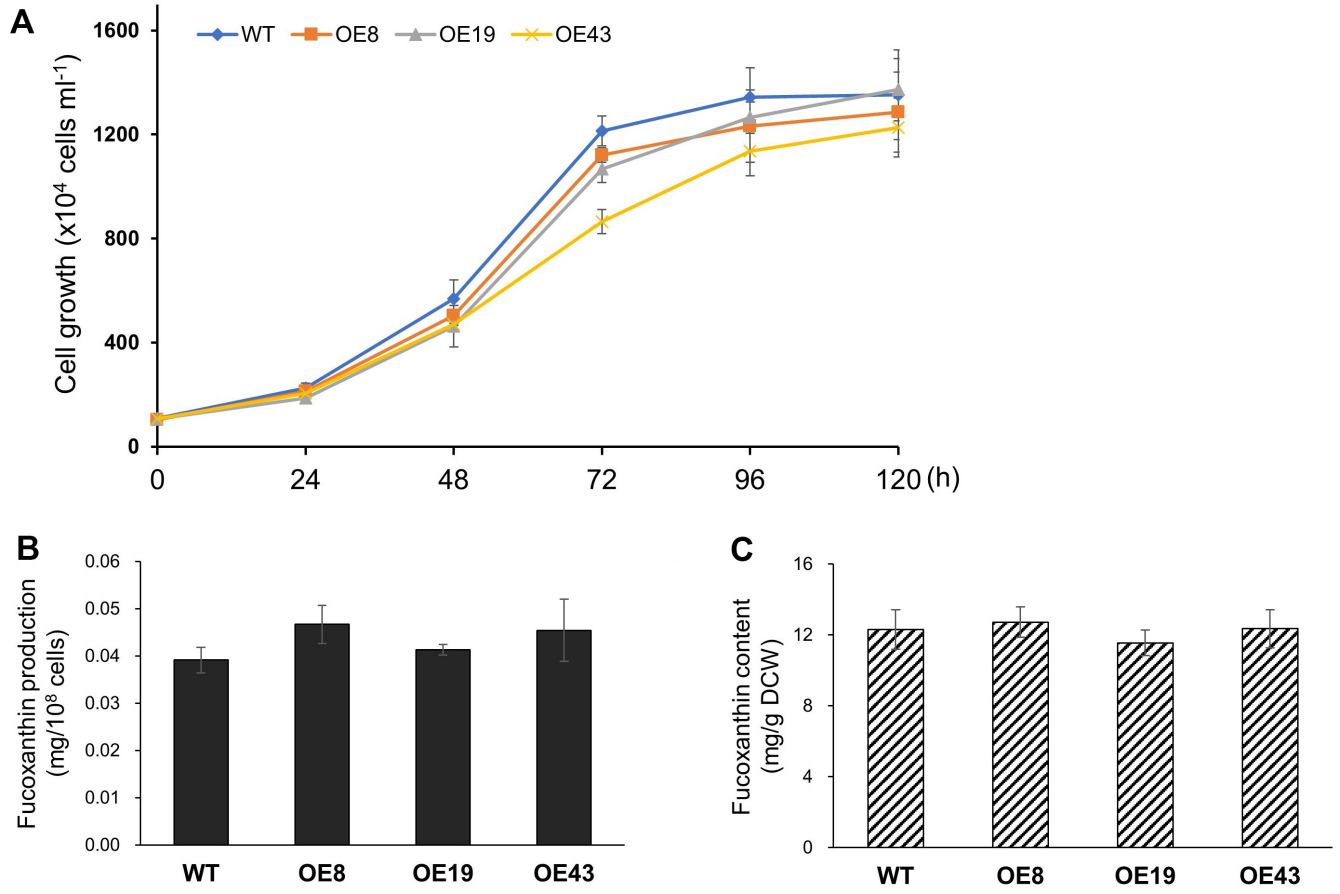
Cell growth and fucoxanthin production under white light exposure. (**A**) Growth curves of the wild-type (WT) and PtVDL1 overexpression strains (PtVDL1-OE8, -OE9, -OE43). Each curve shows the mean±SD from three independent replicates. (**B**) Fx production per cells and (**C**) Fx content per biomass weight are analyzed with the samples at 3 d of subculture. Error bars represent mean values from three independent experiments. Statistically significant differences were determined by Student’s *t*-test (**p < 0.01, *p < 0.05). The fucoxanthin contents were analyzed in the samples at 3 d of subculture. Error bars represent the mean values from three independent experiments. Statistically significant differences were determined by Student's *t*-test.

We investigated the effects of PtVDL1 overexpression on cell growth and changes in intracellular Fx levels under normal light/dark cycle conditions. The WT and transgenic strains were inoculated at the same cell density, approximately 1.0 × 10^6^ cells/ml, and cell growth was measured as cell density. After the initial inoculation, the cells exhibited exponential growth until day 3 of culture. Subsequently, cell growth enters the stationary phase. After 3 d of subculture, the PtVDL1-OE43 strain showed a slight growth delay compared to the WT, although no difference in growth was observed on day 5. Furthermore, on day 5 after inoculation, the cell density of the WT reached 13.53 ± 1.72 × 10^6^ cells/ml, whereas the cell numbers of the transformants PtVDL1-OE8, -OE19, and -OE43 were 12.86 ± 1.54, 13.73 ± 1.20, and 12.26 ± 1.13 × 10^6^ cells/ml, respectively, and no statistically significant differences in growth were observed (Fig. 5A).

In PtVDL1-OE transformants, an increase in PtVDL1 protein resulted in enhanced substrate consumption, thus promoting violaxanthin depletion. To determine whether this resulted in enhanced Fx production, we investigated the Fx content in *P. tricornutum* cultured under normal growth conditions. In the late phase of exponential growth, specifically on day 3 of cultivation, we evaluated the production and content of Fx, measured as weight per cell number and dried cell weight, in the WT and three transformants expressing PtVDL1. According to HPLC analysis, the Fx content in WT *P. tricornutum* was 0.039 mg/10^8^ cells and 12.32 mg/g dried cell weight. In addition, the Fx content of PtVDL1-OEs was not significantly different from that of WT (Fig. 5B and 5C).

### The Effects of Red Light on the PtVDL1 Overexpression

**Fig. 6:**
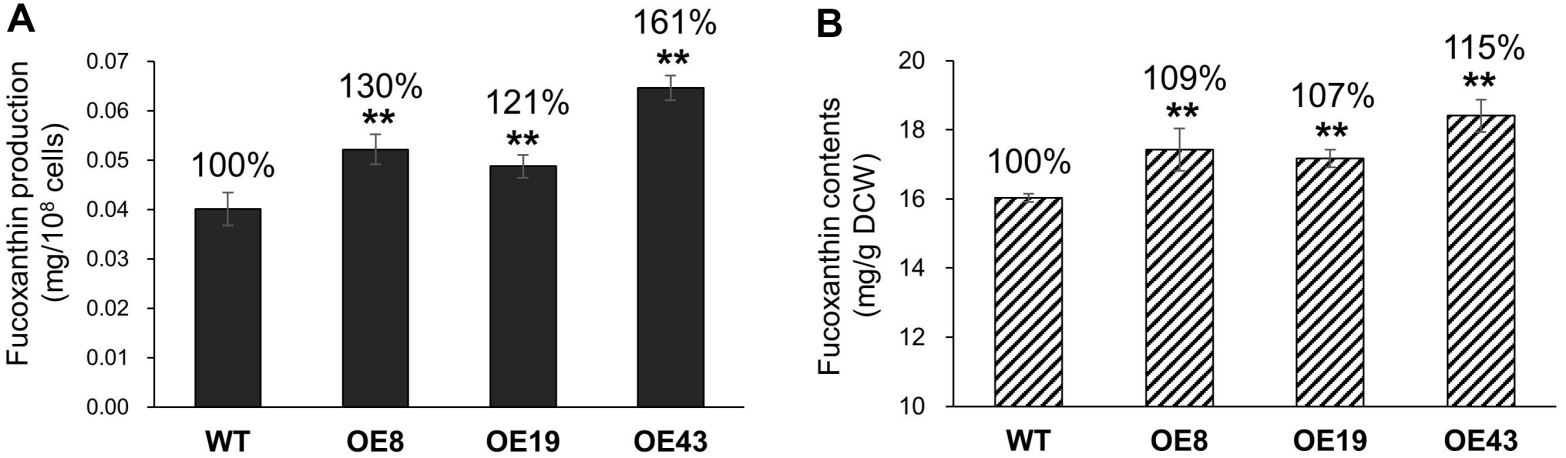
Fucoxanthin production after 48h of exposure to red light. (**A**) Fx production per cells and (**B**) Fx content per biomass weight are analyzed with the cells exposed to red light for 48 h. Error bars represent mean values from three independent experiments. Statistically significant differences were determined by Student’s *t*-test (**p < 0.01, *p < 0.05).

Studies have shown that red light decreases the relative transcript levels of VDL1, whereas *P. tricornutum* cultured under light conditions with longer wavelengths in the red spectral region increases the production of carotenoids such as Fx [21, 22]. Because Fx production in VDL1-overexpressing *P. tricornutum* was slightly altered under normal light conditions (Fig. 5), we examined the changes in the in vivo Fx content of the transformants grown under red-light conditions for 48 h after 3 d of normal light-dark cycles. Transformation of *P. tricornutum* overexpressing PtVDL1 resulted in increased accumulation of Fx compared to the WT under red-light treatment conditions. In PtVDL1-OE8 and -OE19, it was significantly increased by 30% and 21% per cell, respectively. PtVDL1-OE43 showed a 61% significant increase in Fx content per cell, whereas OE8, OE19, and OE43 exhibited 9%, 7%, and 15% significant increases in Fx content per biomass weight, respectively, compared to the WT *P. tricornutum* (Fig. 6A and 6B). These results indicate that the overexpression of PtVDL1 enhances Fx production under red-light illumination.

## Discussion

Diatoms are unicellular phytoplankton of significant importance that obtain their photosynthetic apparatus through two rounds of endosymbiosis and are responsible for approximately 25–40% of the total primary production of marine organisms [12]. Marine diatoms are highly proliferative and can easily be cultivated in diverse marine water culture systems. In addition, their high Fx content makes them important as potential production organisms for commercial applications. Fx biosynthesis in diatoms has been extensively studied in plants, and genes associated with the pathway have been identified as key players in the pathway [23, 24]. However, the underlying biosynthetic pathways are not fully understood. Photosynthetic organisms, including diatoms, utilize the methyl-d-erythritol phosphate (MEP) pathway in plastid organelles to generate isoprenoid precursors IPP (isopentenyl pyrophosphate) and dimethylallyl diphosphate (DMAPP). From these precursors, C20 geranylgeranyl pyrophosphate, the phytoene precursor, is synthesized, followed by a multistep reaction to produce zeaxanthin through phytoene, b-carotene, lycopene, and b-carotene. Zeaxanthin undergoes epoxidation and de-epoxidation through ZEP and VDE, respectively, as a constituent of the xanthophyll cycle, whereas violaxanthin synthesis is controlled. In diatoms, epoxidation occurs under low light conditions, whereas de-epoxidation occurs under more intense light. The epoxidation rate was high under low-light conditions. This promotes the production of violaxanthin, which serves as a precursor for Fx synthesis [25, 26]. The light-dependent xanthophyll cycle serves not only to provide photoprotection under high light but also to increase the production of light-harvesting carotenoids under low light.

Although it is generally accepted that violaxanthin functions as a precursor for Fx production, the genes involved in the multistep Fx biosynthesis in violaxanthin are still in the process of being identified. Diatoms contain multiple copies of xanthophyll cycle-related genes, including those encoding epoxidase and de-epoxidase. In *P. tricornutum*, three genes (PtVDE, PtVDL1, and PtVDL2), which encode isoforms of VDE, have been reported to be located in the genome [21]. Among these, PtVDL1 and PtVDL2 were not involved in de-epoxidation of violaxanthin and diadinoxanthin. Specifically, PtVDL1 was found to facilitate the conversion of violaxanthin to neoxanthin in transient expression experiments in tobacco leaves and in vitro assays [18]. Thus, this study aimed to determine the in vivo role of PtVDL1 by characterizing the effects of its mRNA and protein overexpression in *P. tricornutum*.

Initially, we produced three strains exhibiting high levels of PtVDL1 expression under the strong Fx chlorophyll a/c binding protein B (fcpB) promoter [27]. We then analyzed the cellular content of neoxanthin in the transformants using HPLC and found that neoxanthin was undetectable and violaxanthin levels were significantly diminished compared to those in the WT (Fig. 4; Table 1). Therefore, it was predicted that PtVDL1 plays a critical role in the in vivo conversion of violaxanthin to neoxanthin in *P. tricornutum*. However, neoxanthin does not accumulate within cells because of its rapid conversion to the next metabolite. Neoxanthin is considered an intermediate of Fx in diatoms. Although neoxanthin is present in small amounts in microalgae, particularly diatoms [28], its quantification in *P. tricornutum* through HPLC analysis is highly limited. Specifically, *P. tricornutum* comprises Fx (10.67 mg/g), diadinoxanthin (3.26 mg/g), diatoxanthin (0.20 mg/g), violaxanthin (0.05 mg/g), and zeaxanthin (0.04 mg/g) as measured through HPLC analysis. Neoxanthin was not detected [29].

In this study, we confirmed that the selected PtVDL1 overexpressing transformants maintained high transcript and protein expression levels without any significant effect on cell growth. When the three PtVDL1 overexpressors selected in this way were cultured in a normal white-light environment, changes in Fx content were confirmed, and there was no difference in Fx content per cell or biomass (Fig. 5). This may be because the biosynthetic process of Fx production from neoxanthin consists of several additional steps. These steps involve PtVDL2, a paralog of PtVDL1, which tautomerizes diadionoxanthin to produce allenoxanthin. This is then epoxidized by ZEP1 to produce phaneroxanthin, which is finally hydrated by CRTISO5 to produce Fx in *P. tricornutum* [13, 30]. *AtABA4*, the gene necessary for neoxanthin biosynthesis in *Arabidopsis*, is responsible for the conversion of violaxanthin to neoxanthin. *PtABA4* from *P. tricornutum*, an ortholog of *AtABA4*, showed high homology, and the induction of *PtABA4* overexpression resulted in an increase in Fx in *P. tricornutum* under cool white light. However, in vitro neoxanthin synthase activity was not observed *PtABA4* when was expressed in *E. coli*. It has been suggested that PtABA4 forms multi-enzyme complexes with other membrane proteins, indicating its exclusive involvement in carotenoid formation instead of neoxanthin [29]. Therefore, to enhance Fx biosynthesis, in addition to the overexpression of PtVDL1, the activity of downstream enzymes must be increased.

Previous studies have shown that red light induces downregulation of Fx-related genes, including VDL1 and VDL2. Therefore, the effect of PtVDL1 on Fx biosynthesis could be better characterized by the overexpressing VDL1 compared with WT *P. tricornutum*. This may exacerbate the difference from the WT owing to the overexpression of VDL1 [21]. In addition, pigment composition can be altered by the ratio of red to blue light and a shift to white light. Some studies have shown that blue light is more suitable for Fx production, whereas others have shown that red wavelength light increases Fx content [22, 31] and that low light intensity also increases Fx levels [32]. Red wavelengths have been shown to induce greater production of photoprotective carotenoids, particularly Fx [22], whereas other studies have reported that the Fx content of red light is lower than that produced under conditions of white light [33]. Although the amount of the VDL1 transcript increased over time under red-light conditions, it was expressed at a lower level than that under white light conditions, and the overall expression of other genes involved in carotenoid synthesis was reduced in the red-light environment, which may have contributed to the reduced productivity of Fx [21, 33]. Therefore, to maximize the effect of Fx accumulation upon PtVDL1 overexpression, it was necessary to determine the response under low- intensity red-light exposure conditions. Accordingly, the results showed that PtVDL1 overexpression did not alter Fx levels under white light conditions (normal culture conditions). However, exposure to red light significantly increased the Fx levels (Figs. 5 and 6). The enhanced production of Fx in the overexpression lines of PtVDL1, leading to a greater conversion of violaxanthin to neoxanthin, may be attributed to the stimulation of enzyme activity by red light in the subsequent step after neoxanthin treatment. In the WT, there was no change in Fx content per cell, but Fx content per dry cell weight was increased from 12.3 mg/g to 16.0 mg/g under red light conditions compared to white light (Fig. 5 and 6). These results suggest that the overexpression of PtVDL1 under conditions that induce the production of photoprotective carotenoids, specifically Fx, by exposure to red light, enhances the conversion of violaxanthin to neoxanthin, ultimately leading to the synthesis of Fx.

The light-mediated gene expression might be the key controlling carotenogenesis in photosynthetic organisms. Schofield and Paliyath demonstrated post-translational control of PSY mediated by phytochrome. It was observed that PSY activity is reversibly regulated by red and far-red light as a consequence of phytochrome inactivation and activation, leading to an increase in PSY activity only under red light conditions [34]. Therefore, light, as sensed by photoreceptors, regulates carotenoid biosynthesis through transcriptional and post-transcriptional mechanisms. Recently, it was discovered that two marine diatom species, *P. tricornutum* and *Thalassiosira pseudonana*, possess a unique red/far-red light-sensing phytochrome [35]. In this study, diatom phytochrome displays distinctively red-shifted absorbance peaks compared to other known plant and algal phytochromes. When exposed to both red and far-red light, *P. tricornutum* undergoes changes in gene expression. The VDL1 gene involved in Fx synthesis in *P. tricornutum* may be regulated by phytochrome, similar to what has been observed in carotenoid biosynthesis in green plants [34]; however, further confirmation is needed.

In conclusion, we obtained PtVDL1, a gene involved in Fx synthesis, and generated overexpression transformants of this gene. We confirmed transcript and protein overexpression in these transformants and examined their growth and Fx productivity. PtVDL1 overexpression in *P. tricornutum* promoted violaxanthin consumption, which was expected to promote its conversion to neoxanthin, to the extent that intracellular levels could not be determined. Subsequent measurement of intracellular Fx levels showed that Fx productivity per biomass was increased by up to 15% following PtVDL1 overexpression under red light rather than white light culture conditions. The molecular mechanism of red-light-induced increase in Fx content should be investigated in future studies.

## Supplemental Materials

Supplementary data for this paper are available on-line only at http://jmb.or.kr.



## Figures and Tables

**Table 1:** The quantification of the peak area of neoxanthin and violaxanthin in WT and PtVDL1-overexpressing *P. tricornutum*.

(*n* = 4)	Neoxanthin	Violaxanthin
WT	Not detected	18.14 μg/g (±3.54)
PtVDL1-OE43	Not detected	Not detected
