# Supplementary material for: Overexpression of PtVDL1 in Phaeodactylum tricornutum Increases Fucoxanthin Content under Red Light
Source: J Microbiol Biotechnol. 2023 Oct 20;34(1):198–206. doi: 10.4014/jmb.2309.09018 (PMC10840463; doi:10.4014/jmb.2309.09018)
Supplement: Supplementary file 1 [file jmb-34-1-198-supple.pdf]

J. Microbiol. Biotechnol.
https://doi.org/10.4014/jmb.2309.09018

Supplementary Table and Figures

Supplementary Table 1. List of primers used for genomic PCR, quantitative real-time PCR, and Southern blot analysis.

Genomic PCR analysis

Primer set for PCR of PtVDL1 (VDL1-iFw, VDL1-iRv)	Forward primer	5'-AGTGCGTCAAGGTCCCGAAA-3'
	Reverse primer	5′-CAGTCAATCACGCCGCCTTC-3′
PCR of exogenous FcpB::PtVDL1 (FcpB pro-Fw, VDL1-ins-Rv)	Forward primer	5′- ACAGCGTCATAATTCGCGGTTGC -3′
	Reverse primer	5′- ACGGTGGCTTCCTTGGCGTA -3′
**ITS** (internal transcribed spacer)	Forward primer	5′-TCCGTAGGTGAACCTGCGG-3′
	Reverse primer	5′-TCCTCCGCTTATTGATATGC-3′

Quantitative real-time PCR

PtVDL1	Forward primer	5'- AGTACCACGCTACGCTCGTT -3'
	Reverse primer	5'- TTCCTTGTCACTCGTTGCTG -3'
TATA-box binding protein (TBP, internal reference gene)	Forward primer	5'-TTGCCAGTTACGAGCCAGAG-3'
	Reverse primer	5'-CGCCAGGTCCATTTCCTTCT-3'

Southern blotting

PCR primers for probe	Forward primer	5'-CCACTCTTGACGACACGGCT-3'
	Reverse primer	5'-CGGAGTACGAGACGACCACGA-3'

(A)

```
atg cga ttc gct tgg gtg gta gca gcc gga gtc gtc ttg acg acg acg aca caa gcc ttg   60
 M   R   F   A   W   V   V   A   A   G   V   V   L   T   T   T   T   Q   A   L
gtg cct tta gat tgc acg ggt atg ggt gaa aca aga acg agc ggc atc cgt ccg atc cgg  120
 V   P   L   D   C   T   G   M   G   E   T   R   T   S   G   I   R   P   I   R
agt ctg gaa agc aac atg gcc cgg tac gct act gta cgc cac ggc acc gac cag acg aac  180
 S   L   E   S   N   M   A   R   Y   A   T   V   R   H   G   T   D   Q   T   N
cac ggc ata act tcc tct tct gaa aga cag tgg cct ttt cca cgc ggg ggt tct tct ccc  240
 H   G   I   T   S   S   S   E   R   Q   W   P   F   P   R   G   G   S   S   P
aga gcg gta gcg agg tcc gtg gcg acg ttc gga ctc ggt ttc tcc atc gca ctg gcg agt  300
 R   A   V   A   R   S   V   A   T   F   G   L   G   F   S   I   A   L   A   S
gtt ttc gga gtc gcg gcg gct ccg gtc ggc gcc gac acg act ccc gcc gtc aag tac gac  360
 V   F   G   V   A   A   A   P   V   G   A   D   T   T   P   A   V   K   Y   D
gga ttc gca gaa tac gct caa gat aat caa atg gag caa tct gac gtc gga tgc ttt atc  420
 G   F   A   E   Y   A   Q   D   N   Q   M   E   Q   S   D   V   G   C   F   I
aat aaa tgt ggt gat caa acc aag gca ctc ttt agc aat ccg cgt gga atc aaa ggt gtc  480
 N   K   C   G   D   Q   T   K   A   L   F   S   N   P   R   G   I   K   G   V
tcc tgt ttg gga cgc tgc aag ggc gag caa tcg tgt gcg acg cgg tgt ttt gcc gaa ttc  540
 S   C   L   G   R   C   K   G   E   Q   S   C   A   T   R   C   F   A   E   F
gga agc gag agc tta aac gca tgg ttg tcc tgt act atc gag gaa aac gag tgc gtc aag  600
 G   S   E   S   L   N   A   W   L   S   C   T   I   E   E   N   E   C   V   K
gtc ccg aaa aat gtc gac aac agt gcg gaa gac atc ggc tac agt acc acg cta cgc tcg  660
 V   P   K   N   V   D   N   S   A   E   D   I   G   Y   S   T   T   L   R   S
ttt gat ccg cag agt ttg gtg ggt acg tgg tac aag acc gat gga ttg aat ccc aac tac  720
 F   D   P   Q   S   L   V   G   T   W   Y   K   T   D   G   L   N   P   N   Y
gat ttg ttt gac tgt cag aag aat acc ttt aca gca acg agt gac aag gaa ctc gat atg  780
 D   L   F   D   C   Q   K   N   T   F   T   A   T   S   D   K   E   L   D   M
gga ata ttc ttc cgt gtc caa cga ccg cca gag tcg ggc ggt ggc tat tgg gaa aac gct  840
 G   I   F   F   R   V   Q   R   P   P   E   S   G   G   G   Y   W   E   N   A
ctc acc gaa cac atg atc gta gat gtc ccg gta cag ccc ccg act gcc ggg act caa ctc  900
 L   T   E   H   M   I   V   D   V   P   V   Q   P   P   T   A   G   T   Q   L
gtc gcg tcc gcc aat gcc gcc act ggt gac ctt aac gaa gaa ctc aat ccg acc ggt cgc  960
 V   A   S   A   N   A   A   T   G   D   L   N   E   E   L   N   P   T   G   R
acc atg cat acc gcc ggt aaa atg tac ggt ctc gag ttt acc gaa aac tgg tac att ctc 1020
 T   M   H   T   A   G   K   M   Y   G   L   E   F   T   E   N   W   Y   I   L
ggt gaa tcc gac ggt aag gga tcg gtt ccc cct ttc aag ctc gtg gca tac aag ggc cac 1080
 G   E   S   D   G   K   G   S   V   P   P   F   K   L   V   A   Y   K   G   H
aca ttg caa ggc aac tac gaa ggc gcc ttt gtg tac gcc aag gaa gcc acc gtg ccg gaa 1140
 T   L   Q   G   N   Y   E   G   A   F   V   Y   A   K   E   A   T   V   P   E
gct gcc aaa cca gcg att cgc gag gct gcc acc aag gcg ggc ttg gat ttt gat gcc ttt 1200
 A   A   K   P   A   I   R   E   A   A   T   K   A   G   L   D   F   D   A   F
act cgt atc gac aat acc tgt tcg gta gga gac tct ttg aac gat gca caa gcg ggg acc 1260
 T   R   I   D   N   T   C   S   V   G   D   S   L   N   D   A   Q   A   G   T
gga acg tcc acg aca gat tgg atc aat ctc gtc gtc ggt gaa ggc ggc gtg att gac tgg 1320
 G   T   S   T   T   D   W   I   N   L   V   V   G   E   G   G   V   I   D   W
att tct ccc gga tgg cgg ggg gaa tac aag gcg aaa cgc cat atg gaa caa aaa ctc att 1380
 I   S   P   G   W   R   G   E   Y   K   A   K   R   H   M   E   Q   K   L   I
agt gaa gaa gat ctt taa 1398
 S   E   E   D   L   *
   c-myc tag
```

(B)

```
PtVDL1          1   MRFAWVVAAGVVLTTTTQALVPLDCTGMGETRTSGIRPIRSLESNMARYATVRHGTDQTN    60
XP_002180635    1   MRFAWVVAAGVVLTTTTQALVPLDCTGMGETRTSGIRPIRGLESNMARYATVRHGTDQTN    60

PtVDL1         61   HGITSSSERQWPFPRGGSSPRAVARSVATFGLGFSIALASVFGVAAAPVGADTTPAVKYD   120
XP_002180635   61   HGITSSSERQWPFPRGGSSPRAVARSVATFGLGFSIALASVFGVAAAPVGADTTPAVKYD   120

PtVDL1        121   GFAEYAQDNQMEQSDVGCFINKCGDQTKALFSNPRGIKGVSCLGRCKGEQSCATRCFAEF   180
XP_002180635  121   GFAEYAQDNQMEQSDVGCFINKCGDQTKALFSNPRGIKGVSCLGRCKGEQSCATRCFAEF   180

PtVDL1        181   GSESLNAWLSCTIEENECVKVPKNVDNSAEDIGYSTTLRSFDPQSLVGTWYKTDGLNPNY   240
XP_002180635  181   GSESLNAWLSCTIEENECVKVPKNVDNSAEDIGYSTTLRSFDPQSLVGTWYKTDGLNPNY   240

PtVDL1        241   DLFDCQKNTFTATSDKELDMGIFFRVQRPPESGGGYWENALTEHMIVDVPVQPPTAGTQL   300
XP_002180635  241   DLFDCQKNTFTPTSDKELDMGIFFRVQRPPESGGGYWENALTEHMIVDVPVQPPTAGTQL   300

PtVDL1        301   VASANAATGDLNEELNPTGRTMHTAGKMYGLEFTENWYILGESDGKGSVPPFKLVAYKGH   360
XP_002180635  301   VASANAATGDLNDELNPTGRTMHTAGKMYGLEFTENWYILGESDGKGSVPPFKLVAYKGH   360

PtVDL1        361   TLQGNYEGAFVYAKEATVPEAAKPAIREAATKAGLDFDAFTRIDNTCSVGDSLNDAQAGT   420
XP_002180635  361   TLQGNYEGAFVYAKEATVPEAAKPAIREAATKAGLDFDAFTRIDNTCSVGDSLNDAQAGT   420

PtVDL1        421   GTSTTDWINLVVGEGGVIDWISPGWRGEYKAKR   453
XP_002180635  421   GTSTTDWINLVVGEGGVIDWISPGWRGEYKAKR   453
```

Supplementary Figure 1. Characterization of sequences of PtVDL1 gene in *Phaeodactylum tricornutum*.

(A) The full length cDNA sequence and its deduced amino-acid sequence of PtVDL1 and c-myc tag. The translation of the coding regions is also given and the stop codon is indicated by an asterisk. Yellow-filled box is c-myc tag. **(B)** Alignment of PtVDL1 protein sequences with PtVDL1 from *P. tricornutum* CCAP 1055/1 (XP_002180635).
